# Is Electricity an Anæsthetic?

**Published:** 1859-12

**Authors:** 


*Is Electricity au Anæsthetic?*—The question has been raised in the College of Dentists of England. At a late meeting, Mr. Peter Matthews, President of the College, occupied the chair. He opened the debate with an address on the various anæsthetic agents used in surgery, and ultimately came to the question: Is electricity an anæsthetic, or is it not? He (the President), after a long and careful experience, had no alternative left but to answer that question in the negative. He then related with great precision the details of various cases of extraction of teeth, in which he had employed galvanism, but explained that although the result was, in some instances, modified from ordinary tooth-drawing, he could not admit that in any instance had the pain of extraction been abolished. In certain peculiar cases, indeed (cases where the elastic tissue which connects the tooth with its socket, the tooth periosteum, is inflamed and painful), the application of galvanism in extraction adds to the pain of the operation. Mr. Matthews then described the different modes of applying the electric current, and concluded by expressing his opinion that, in our present state of knowledge, electricity could not be considered as an anæsthetic agent. Dr. Purland, Mr. Weiss, Mr. Lobb, and Mr. Perkins, joined in the discussion, the tenor of their experience being consonant with that of the President. It was clearly the general feeling, that galvanism in tooth extraction, acts only by producing a diversion of pain, not by causing insensibility.—*Nashville Monthly Record.*

